# Effect of postpartum depression, anxiety and social support on maternal self-efficacy: comparing undocumented migrant, documented migrant and Swiss-born mothers

**DOI:** 10.3389/fpsyg.2025.1477817

**Published:** 2025-03-12

**Authors:** Anna Sharapova, Betty Goguikian Ratcliff

**Affiliations:** ^1^Hôpitaux Universitaires de Genève (HUG), Geneva, Switzerland; ^2^Faculté de Psychologie et des Sciences de l’Éducation, Université de Genève, Geneva, Switzerland

**Keywords:** postpartum depression, anxiety, maternal self-efficacy, migrant, legal status

## Abstract

**Introduction:**

Postpartum depression and anxiety negatively affect maternal sense of self-efficacy, which may jeopardize mother-infant bonding. Migrant women are at two to three times higher risk for postpartum depression and anxiety. Therefore, they may experience lower maternal self-efficacy, but studies on the subject are lacking. The aims of this study were (1) to compare two groups of economic migrants of differing legal status in Geneva, Switzerland, to native Swiss women in terms of postpartum depression and anxiety rates, as well as maternal sense of self-efficacy, and (2) to examine the effects of postpartum depression, anxiety, and social support on maternal self-efficacy in the three groups.

**Methods:**

A sample of 25 undocumented migrant women, 42 documented migrant women, and 41 Swiss women were interviewed at 3 months postpartum. Depression was assessed with the Edinburgh Postpartum Depression Scale and anxiety with the State–Trait Anxiety Inventory. Maternal self-efficacy was assessed with the Maternal Self-Efficacy Scale. ANOVAs and multiple regression analysis were used to test mean-level differences among the three groups and identify factors associated with low maternal self-efficacy.

**Results:**

Legal status was associated with living conditions and influenced the rates of postpartum distress. Swiss women and documented migrant women showed low depression and anxiety rates, whereas nearly half of the sample of undocumented women reported high levels of postpartum depression and anxiety. However, despite poor postpartum mental health, undocumented women showed a higher sense of maternal self-efficacy than did documented migrants and Swiss natives. The relationship between postpartum depression, maternal self-efficacy, social support, and legal status is discussed.

## Introduction

Postpartum depression is recognized as a major public health issue (World Health Organization, 2009), with about 10–15% of women presenting depressive symptoms in the postpartum period (Shorey et al., 2018; Liu et al., 2022). Numerous specific symptoms, such as low maternal self-efficacy (feeling unable to take care of the baby), lack of concern for the baby, guilt, and acute anxiety characterize depression related to childbirth and highlight its specific nature (Patel et al., 2012; Brummelte and Galea, 2016).

Postpartum anxiety demonstrates a greater prevalence rate than depression but appears to be under-recognized by perinatal health care professionals and has received limited attention from researchers (Leach et al., 2015; Dennis et al., 2017). Mothers’ frequent postpartum worries concern the baby’s development and health, changes in the relationship with her partner, housing, giving up work, and financial problems (Rowe et al., 2014). Women who lack personal, social, or financial resources, those who have psychiatric comorbidities or who have been previously exposed to adverse life events, may experience exacerbated anxiety (Falah-Hassani et al., 2016; Dennis et al., 2018).

A growing body of research highlights the pervasive effects of postpartum depression and anxiety on maternal self-efficacy (Kohlhoff and Barnett, 2013; Kunseler et al., 2014). Maternal self-efficacy refers to the mother’s own beliefs in her ability to respond to her baby’s needs and to deal with stressful situations related to motherhood (Bandura, 1997; Leahy-Warren and McCarthy, 2011). Maternal self-efficacy has been linked to competent parenting practices (Leahy-Warren and McCarthy, 2011), positive mother–child relationships (Abarashi et al., 2014), optimal child development (Léniz-Maturana et al., 2022), and secure attachment in the child (Cassé et al., 2016). Postnatally depressed mothers may have difficulty interpreting their child’s demands, may show less pleasure in parenting, and are less responsive to their infant’s needs (Field, 2010; Stein et al., 2014). They report a lack of confidence in the mother’s role and an inability to care for the infant (Leahy-Warren et al., 2012; Brummelte and Galea, 2016). Low maternal self-efficacy may result in a disrupted mother–child relationship and affect the infant’s cognitive and affective development (Field, 2010; Léniz-Maturana et al., 2022).

### Migrant women and transition to motherhood

Migrant women are two to three times at higher risk for postpartum depression than are native women (Dennis et al., 2018; Xiong and Deng, 2020; Zlotnick et al., 2022). They also seem to be at higher risk for postpartum anxiety (Dennis et al., 2017). These important rates of postpartum distress have been associated with various environmental stressors related to the migration experience. The lack of local language proficiency, inappropriate housing, socioeconomic difficulties, cultural differences, separation from family, and social isolation have been identified as major risk factors for postpartum depression and anxiety in migrant women (Fung and Dennis, 2010; Dennis et al., 2017; Xiong and Deng, 2020; Zlotnick et al., 2022). Undocumented (Davoudian, 2012; Jackson et al., 2018) and asylum seeking (Goguikian Ratcliff et al., 2015a; Mestre, 2016) women face cumulative psychosocial difficulties and are at particularly high risk for perinatal depression and anxiety. The absence or uncertainty of a legal status during the perinatal period is a highly significant risk factor.

In Western psychology, parenting and early parental care have been conceptualized in terms of inner parental characteristics such as maternal self-efficacy. Yet, this approach is culturally loaded and embedded in Western individualistic representations of parenting. Parenting practices widely differ from collectivistic to individualistic cultures (Yaman et al., 2010). In collectivistic lifestyle, the presence and help of elder female relatives is of major importance during the postpartum period. In these societies, the mother’s new social status is celebrated and marked with various protective rituals and taboos (Team et al., 2009; Moro et al., 2013). When migrant women coming from non-Western, rather collectivistic societies give birth in Western countries, they are confronted to an individualistic cultural frame, where the baby is often the primary focus of attention, and where maternity is perceived as an individual responsibility of the mother or the couple. The lack of cultural postpartum rituals and family guidance can lead to a sense of maternal incompetence among migrant new mothers (Baubet and Moro, 2013; Goguikian Ratcliff and Diaz-Marchand, 2019). Studies have also shown that lack of social support (Mestre, 2016; Xiong and Deng, 2020) and precarious living conditions (Leahy-Warren et al., 2012) may impinge on maternal self-efficacy.

### Aims of the study and hypotheses

Previous considerations emphasize the importance of separate screening for depression and anxiety in the postpartum period. Early identification and treatment of these disorders may help to prevent their impact on the mother, the child, and their relationship (Brummelte and Galea, 2016; Dennis et al., 2017). Studies on postpartum anxiety, depression, and maternal self-efficacy among migrant women have been limited. Moreover, existing studies on postpartum distress mostly focused on refugees and forced migration, while undocumented economic migrants have received less attention. Our study focused on voluntary economic migration and did not include asylum seeking women nor refugees. Refugee mothers often report exposure to pre-migration traumatic events, and present high prevalence of Post-Traumatic Stress Disorder (PTSD) (Daou, 2022). In the present study, we chose to focus on legal status and its association with depressive symptoms after childbirth. Moreover, few studies on the subject included a sample of high-income documented migrant women and a control sample of native women.

In Switzerland, nearly two thirds of permanent foreign nationals are documented migrants who come from Western Europe and other Organisation for Economic Co-operation and Development (OECD) countries (Federal Statistical Office, 2017a). Most of them are highly educated, moving to Switzerland for working reasons. They benefit from a long-term residency permit and receive social rights. On the other hand, around 1% of the Swiss population is represented by undocumented migrants, half of whom are women (Morlok et al., 2015; Federal Statistical Office, 2017b). Most of these undocumented migrants come from Latin America, non-EU countries, Asia or North/West Africa (Federal Commission on Migration (FCM), 2024). Most of them work in care services and endure difficult living conditions (Morlok et al., 2015), as they do not have access to basic social services and healthcare.

The present study aimed to compare two groups of economic migrant women of differing legal status (documented vs. undocumented) and socioeconomic conditions with native Swiss women in relation to postpartum depression and anxiety rates, as well as maternal sense of self-efficacy and social support. It further aimed to compare the effects of postpartum depression, anxiety, and social support on maternal self-efficacy among the three groups. In the light of the previous considerations, we hypothesized that, compared with Swiss women, migrant women, in particular undocumented women, would report higher depression and anxiety rates and lower maternal self-efficacy. We also expected that postpartum depression and anxiety would predict low maternal self-efficacy, whereas social support would predict high maternal self-efficacy in all groups.

## Materials and methods

### Sample and procedures

Simple random sampling was used for the study. Swiss women and documented migrant women were recruited during pregnancy through flyers and in person while attending birth preparation classes at the Maternity Hospital and a collective of independent midwives. Undocumented migrant women were recruited in person during free medical tests performed during pregnancy at the Maternity Hospital. Due to recruitment challenges, particularly in reaching the undocumented group, no formal power analysis was conducted prior to data collection. The sample size was determined by practical constraints, including the limited recruitment timeframe and the availability of participants. Women who agreed to participate were contacted by phone to set up a postpartum interview during the third month after the delivery. Interviews were conducted by the main author between January 2015 and February 2018. This study is part of a larger longitudinal research project on perinatal mental health in migrant women (Sharapova and Goguikian Ratcliff, 2018; Sharapova and Goguikian Ratcliff, 2021).

Migrant women were defined as those who were born outside of Switzerland and who migrated during adulthood. Undocumented migrant women were those who had no legal residential permit in Switzerland, while documented migrant women were those who had a long-term residency permit.

The inclusion criteria were as follows: third month postpartum, aged 18 years and older, and able to respond to questions and to complete questionnaires in English, French, Spanish, or Portuguese. The exclusion criterion was receiving follow-up care for a severe psychiatric disorder.

The sample consisted of 108 women: 25 (23%) undocumented migrants, 42 (39%) documented migrants, and 41 (38%) Swiss natives. The study procedure was approved by the Faculty of Psychology and Educational Sciences Ethical Committee at the University of Geneva. All women provided written consent and were informed that clinical information would be codified and treated anonymously. All participants were informed that they could withdraw their participation at any time without any consequences. Women who presented clinical anxiety and depression scores were oriented to specific psychiatric centers, either those working with basic health insurance, or those receiving undocumented migrants with no insurance.

### Measures

A 21-item questionnaire was used to obtain sociodemographic information (age, nationality, education level, date of arrival in Geneva, legal status, French proficiency) and psychosocial living conditions (housing, work rate, presence of family members and friends in Geneva).

*The Edinburgh Postpartum Depression Scale (EPDS)* (Cox et al., 1987) was used to screen for postpartum depression. It is a 10-item self-report instrument largely used in international perinatal research (Cox et al., 2014). Responses are scored 0, 1, 2 and 3 based on the seriousness of the symptom (e.g., “I have felt sad or miserable”). The total score is found by adding together the scores for each of the 10 items. Higher score indicates stronger symptom severity. In the present study, a cutoff of 10 was applied, as recommended by the authors when the scale is used as a unique measure of postpartum depressive symptoms (Cox et al., 1987). Furthermore, Levis et al. (2020) suggested using lower cut-off values than frequently applied cutoff of 13 to avoid false negatives and identify most patients who meet diagnostic criteria. Translated and validated versions of the scale in French (Guedeney and Fermanian, 1998), Spanish (Garcia-Esteve et al., 2003), and Portuguese (Tendais et al., 2014) were used for this study. Cronbach’s alpha for this scale was 0.77.

*The State–Trait Anxiety Inventory (STAI)* (Spielberger, 1983), particularly the state anxiety subscale, was used to assess anxiety as an emotional state. The subscale consists of 20 items (e.g., “I feel nervous”), and each item is scored on a 4-point scale, with overall scores varying from 20 to 80. Anxiety is considered low up to a score of 45, moderate between 46 and 55, and high when the score is 56 or more. For this study, translated and validated versions of the scale in French (Bruchon-Schweitzer and Paulhan, 1993), Spanish (Buela-Casal et al., 2011), and Portuguese (Tendais et al., 2014) were used. The state anxiety subscale has shown very good internal consistency, with a Cronbach’s alpha of 0.91.

*The Short-Form Social Support Questionnaire* (Sarason et al., 1987) was used to assess social support. The scale allows to measure two dimensions: the extent of social support, i.e., the number of people available for emotional or practical support (e.g., “With whom can you totally be yourself?”), and the degree of satisfaction with the available support (the quality of support), scored on a 6-point scale (score ranging from 6 to 36 points). Higher score indicates higher social support. For this study, translated and validated versions of the scale in French (Bruchon-Schweitzer et al., 2003), Spanish (Friedman et al., 2018), and Portuguese (Matsukura et al., 2007) were used. Cronbach’s alpha was 0.91 for the extent of social support and 0.89 for the quality of support.

*The Maternal Self-Efficacy Scale (MSES)* (Teti and Gelfand, 1991) is a frequently used self-report instrument that consists of nine items that assess women’s feelings regarding efficacy in relation to specific domains of infant care, such as interpreting baby’s demands, performing routine tasks, and disengaging from the baby (e.g., “How good do you feel you are at feeding, changing, and bathing your baby?”). The 10th item assesses mothers’ global sense of efficacy in mothering. Ratings on each item ranged from 1 (not good at all) to 4 (very good). Higher score indicates higher sense of maternal self-efficacy. Internal consistency of the scale was 0.71 for this sample.

The scores of all scales were used in their continuous form for the analyses.

### Cultural considerations

The mentioned above study languages (English, French, Spanish, Portuguese) were those spoken by the researchers. Validated versions of all standardized questionnaires (EPDS, STAI, SSQ-SF) were used in each language. As for the MSES, it was translated and adapted into French, Spanish, and Portuguese by bilingual psychologists, and then back translated into English to ensure the accuracy of translations. Items requiring construct equivalence brainstorming were discussed within the research team.

### Data analysis

Chi-square tests and t-tests were performed to compare rates for categorical variables and means for continuous variables. We then used ANOVAs to test mean-level differences among the three groups for the EPDS, STAI, and MSES. Correlation analysis was conducted to examine the relationship between postpartum anxiety and depression. Multiple regression analyses were performed for each group to test the impact of postpartum depression, anxiety, social support, and multiparity on maternal self-efficacy. All analyses were conducted with a significance threshold of *α* = 0.05, two-tailed. In the exploratory data analyses, no problems were noted for the assumptions of normality and homogeneity of variance of the variables of interest. The EPDS, the STAI, and the MSES scores were normally distributed (skewness <1, kurtosis <1). There was no missing data as all questionnaires have been completed in the presence of a researcher. There were two outliers who were included in the analyses as they did not affect the results and assumptions.

## Results

### Sample characteristics

All participants ranged in age from 21 to 42 years (*M* = 32.7, *SD* = 4.3). The majority of Swiss women were married or in a couple relationship (*n* = 40, 98%) and had given birth to their first child (*n* = 27, 66%). For 83% of them (*n* = 34), this pregnancy had been planned. All of them had completed secondary or tertiary education and the majority were employed (*n* = 36, 88%) (Table 1).

**Table 1:** Sociodemographic profiles of undocumented and documented migrant vs. Swiss women.

Variable	Undocumented migrants	Documented migrants	Swiss women	ANOVA
	(*n* = 25)	(*n* = 42)	(*n* = 41)	*p*-Value
Mean age (*SD*)	30.8 (6.3)	33.5 (4.2)	31.8 (4.4)	0.053
Civil status: *n* (%)				<.001
Married	9 (36)	36 (86)	23 (56)	
Couple	15 (60)	4 (9)	17 (41)	
Single	1 (4)	2 (5)	1 (3)	
Unintended pregnancy: *n* (%)	18 (72)	3 (7)	7 (17)	<.001
Primiparity: *n* (%)	12 (48)	37 (89)	27 (66)	<.001
Education: *n* (%)				<.001
Primary	5 (20)	1 (2)	0	
Secondary	14 (56)	1 (2)	12 (30)	
Tertiary	6 (24)	40 (95)	29 (70)	
Employment: *n* (%)	25 (100)	32 (77)	36 (88)	0.006
Collective housing: *n* (%)	8 (32)	0	0	<.001
Social support, mean (*SD*)				
Number of people available	3 (1.7)	5 (2)	5.5 (2)	<.001
Satisfaction score (0-36)	27 (2.9)	31 (4.2)	33 (2.9)	<.001
Marital support				
Partner’s presence: *n* (%)	22 (88)	41 (97)	40 (98)	0.021
Mean satisfaction score (*SD*)	8.1 (2.3)	8.8 (1.28)	8.4 (1.25)	0.161
Family presence in Geneva *n* (%)	9 (36)	3 (7)	36 (88)	<.001
Area of origin: *n* (%)				
Western Europe		18 (43)		
Eastern Europe	2 (8)	10 (24)		
Non-European OECD^*^ countries		10 (24)		
Asia	7 (28)	2 (5)		
North/West Africa	5 (20)	1 (2)		
Latin America	11 (44)	1 (2)		
French proficiency				
None	13 (52)	17 (40)		
Limited		5 (12)		
Fluent	12 (48)	20 (48)		

^*^OECD, Organization for Economic Cooperation and Development. Countries concerned here are the United States, Canada, Australia, and Japan.

The profiles of documented migrant women were not significantly different from those of Swiss women in terms of age, education level, social support, or employment rates (Table 1). They were more often married (χ^2^ = 13.42, *p* < 0.01) and primiparas (χ^2^ = 6.84, *p* < 0.05). They were less likely than Swiss women to have family members in Geneva (χ^2^ = 55.13, *p* < 0.001). They had been living in Switzerland for an average of 5 years (*SD* = 3.9). All of them had a long-term residence permit or a work permit.

Among undocumented migrant women half of them (*n* = 13, 52%) were multiparous. For most of them (*n* = 18, 72%), this pregnancy was unintended. Compared to Swiss women, they had a lower educational level (χ^2^ = 57.85, *p* < 0.001), more often lived in collective housing (χ^2^ = 21.9, *p* < 0.001), and reported less social support (*t* = −5.96, *p* < 0.001) (Table 1). They had been living in Switzerland for an average of 3.8 years (*SD* = 3.2). All of them worked part-time or full-time in domestic or care services. Although undocumented migrant women reported poorer social support than documented migrant women did, they had more family members in Geneva (*t* = −3.56, *p* < 0.001).

### Postpartum depression and anxiety

Postpartum anxiety significantly correlated with postpartum depression (*r* = 0.70; *p* < 0.001) in all groups. Regarding depression rates, 10% (*n* = 4) of Swiss women and 12% (*n* = 5) of documented migrant women presented scores equal to or higher than the cutoff point of 10 on the EPDS. Concerning anxiety rates, 12% (*n* = 5) of Swiss women and 10% (*n* = 4) of documented migrant women showed scores higher than 45 on the STAI state anxiety subscale. Therefore, contrary to our expectations, documented migrant women did not significantly differ from Swiss women in terms of the mean scores obtained on the EPDS (*p* = 0.127) and STAI scales (*p* = 0.923).

Undocumented migrant women presented more postpartum depression (*t* = 3.95, *p* < 0.001) and anxiety symptoms (*t* = 3.39, *p* < 0.01) than did Swiss women (Table 2). In fact, 44% (*n* = 11) of them showed clinical scores on the EPDS, and 40% (*n* = 10) reported high scores on the STAI state anxiety subscale.

**Table 2:** Mean scores comparison between the groups on clinical scales.

Scale	Undocumented migrants (*n* = 25)	Documented migrants (*n* = 42)	Swiss women (*n* = 41)	ANOVA *p*-Value
EPDS mean (SD)	11.5 (0.8)	5.8 (3.3)	5.7 (4.8)	<0.01
STAI mean (SD)	46 (8.9)	32 (9.5)	33 (10.3)	<0.01
MSES mean (SD)	4.4 (0.4)	4 (0.3)	4 (0.3)	<0.01

EPDS, Edinburgh Postnatal Depression Scale; STAI, Spielberger State–Trait Anxiety Inventory; MSES, Maternal Self-Efficacy Scale.

### Maternal self-efficacy

Documented migrant women reported high maternal self-efficacy mean scores, similar to those of Swiss women. However, undocumented migrant women reported a significantly higher sense of maternal self-efficacy than did documented migrant women (*t* = − 2.67, *p* < 0.01) and Swiss women (*t* = 2.67, *p* < 0.01) (Table 2).

In Swiss women, maternal self-efficacy was predicted by postpartum depression (*b* = −0.44, *p* < 0.01), but not postpartum anxiety, social support, nor multiparity. A similar pattern was observed in documented migrant women, as maternal self-efficacy was predicted only by postpartum depression (*b* = −0.27, *p* < 0.05). In undocumented migrant women, maternal self-efficacy was not predicted by any of the variables (Table 3).

**Table 3:** Multiple regression results for depression, anxiety, social support, and multiparity as predictors of maternal self-efficacy in migrant and Swiss women.

Group	Predictor	b* ^a^	*SE* ^b^	*t*	*p*	*R2/Adjusted R2*
Swiss women (41)	Depression	−0.44	0.131	−0.2.59	0.003**	0.54/0.49
	Anxiety	−0.12	0.091	−1.32	0.191	
	Social support	0.15	0.089	1.68	0.092	
	Multiparity	0.03	0.093	0.34	0.732	
Documented migrants (42)	Depression	−0.27	0.112	−2.24	0.034*	0.61/0.56
	Anxiety	−0.05	0.081	−0.62	0.124	
	Social support	0.10	0.078	1.28	0.201	
	Multiparity	0.08	0.087	0.92	0.376	
Undocumented migrants (25)	Depression	−0.003	0.004	−0.83	0.415	0.31/0.21
	Anxiety	−0.04	0.045	−0.89	0.382	
	Social support	0.06	0.065	0.92	0.361	
	Multiparity	0.02	0.074	0.27	0.793	

a Standardized regression coefficients.

b Standard errors of b*..

*p < 0.05; **p < 0.01.

Number of observations for each group in parentheses.

## Discussion

The distinctive feature of this study is the consideration of the role of legal status on the mental health of migrant women after childbirth. The two groups of migrant women were represented by two different profiles of women: on the one hand, documented, highly educated and mostly high-income migrant women, and on the other hand, undocumented migrant women with lower educational level who faced considerable employment and housing difficulties. Documented migrant women mostly came from Western European countries, whereas undocumented migrant women mostly arrived from Latin American countries. Both migrant groups dealt with sociocultural factors like the adaptation to a new environment, but only the undocumented migrant group dealt with socioeconomic risk factors; thus, the comparison of these two groups to the group of Swiss women allowed to disentangle environmental factors from sociocultural factors (migration *per se* and adaptation to a new environment).

The rates of postpartum depression and anxiety in the current sample of Swiss women, were similar to those reported in other studies (Dennis et al., 2017; Dennis et al., 2018; Shorey et al., 2018). Contrary to our expectations, documented migrant women in our sample reported depression and anxiety rates that were comparable to those of Swiss women, which are much lower than those reported in other studies on migrant samples (Goguikian Ratcliff et al., 2015a; Dennis et al., 2018). This difference might be explained by the particularity of the present sample of documented migrant women who benefitted from a solid social network and support. Most of them had been living in Geneva for several years, were working, and could freely travel within the European Union to visit their families. Further, most of documented migrant women in our sample were high-income women who had excellent living conditions and access to healthcare. Numerous studies have demonstrated that lack of social support, financial difficulties and lack of access to care represent major risk factors for postpartum depression (Xiong and Deng, 2020; Zlotnick et al., 2022).

Similarly to other studies on postpartum depression in migrant women, undocumented migrant women in our sample showed significantly higher postpartum depression and anxiety scores than did documented women and native Swiss women. These results point to the major role of environmental risk factors in the development of postpartum distress. It seems that migration, as a sociocultural transition, does not necessarily represent a risk factor for postpartum depression and anxiety, but the insecurity of legal status and the cumulative effect of psychosocial, legal, and socioeconomic burden related to the illegal migration make undocumented migrant women extremely vulnerable to postpartum distress (Davoudian, 2012; Jackson et al., 2018). Therefore, the consideration of social, political and cultural context in which psychological distress occurs is crucial, as the nature of the context itself is central to how people respond to, comprehend, and recover from distress. In that sense, anxiety and/or depression symptoms cannot be completely understood by investigating individual characteristics, but the context of their manifestation (Drozdek, 2013).

We expected that migrant women, particularly undocumented ones, would experience a lower sense of maternal self-efficacy than native women because of the lack of social support and higher environmental stressors and cultural discrepancies. It has been shown that the lack of cultural postpartum rituals and family guidance, added to feelings of maternal isolation, can lead to a sense of maternal incompetence among migrant mothers (Baubet and Moro, 2013; Goguikian Ratcliff and Diaz-Marchand, 2019). However, the results showed that undocumented migrant women reported higher maternal self-efficacy than did Swiss women and documented migrant women. One possible explanation for this counterintuitive result is the origin of migrant women in the undocumented group. The majority of them came from Latin American countries, which are mostly collectivist societies where motherhood is perceived as a group matter and children are raised and educated in close interaction with the extended family (Team et al., 2009; Eberhard-Gran et al., 2010; Moro et al., 2013). Thus, most of women in the undocumented group might have already taken part in childrearing in their country of origin. In addition, half of the sample of undocumented women had already given birth to a child. Literature has shown that multiparity is associated to maternal self-confidence (Leahy-Warren and McCarthy, 2011). In the present study, multiparity did not directly predict a higher sense of maternal self-efficacy, but it might have contributed to higher scores on the Maternal Self-Efficacy Scale. Furthermore, maternal self-efficacy did not seem to be perceived as a concern, in comparison to uncertainty and financial difficulties.

Postpartum anxiety was not related to maternal self-efficacy in this study. Postnatally anxious mothers might have engaged in more controlling parenting, thus largely fulfilling all necessary tasks related to childcare. Anxiety did not seem to undermine a mother’s feeling of confidence like depression did.

Postpartum depression predicted low maternal self-efficacy in native Swiss women and documented migrant women, which is consistent with the results of other studies (Kohlhoff and Barnett, 2013; Kunseler et al., 2014). However, this effect was not observed in undocumented migrant women. That is, despite the high rates of postpartum anxiety and depression in this group, women reported high maternal self-efficacy. Similarly, in our previous study on perinatal depression in disadvantaged newcomers in Geneva (Goguikian Ratcliff et al., 2015a), maternal sensitivity toward the baby was assessed through mother-infant interaction observations by a psychologist. The results revealed that, despite the precarious legal status, cumulative environmental stressors, and poor mental health, the majority of migrant mothers showed high maternal sensitivity. It may be hypothesized that the burden of long-term uncertainty and cumulative environmental stressors during the postpartum period, associated with the lack of socioeconomic resources, may result in depression (feeling of helplessness) and anxiety (chronic worries about the future) of an exogenous nature, without necessarily affecting maternal sense of self-efficacy. It has been shown that non-Western mothers do not consider postpartum depression as an illness affecting the mother’s own self-esteem. They describe their distress after childbirth as a product of social stress, largely due to lack of practical support and life difficulties (Gardner et al., 2013; Goguikian Ratcliff et al., 2015b). In contrast, depression in Western mothers is usually explained by inner factors, such as failure to reach personal goals and loss of control, thus affecting the mother’s mood, thoughts and self-esteem (Lehti et al., 2010). The lack of significant direct effect of postpartum depression on maternal self-efficacy in non-Western mothers should be replicated in further studies.

The high reported maternal self-efficacy in undocumented migrant women might also be due to the particularly strong bond between these mothers and their babies, often described as the unique source of stability, joy, and hope. Giving birth during the early stages of migration process implies rapid adjustment to the local norms and educational practices, as well as knowledge of the healthcare system. The contact with the maternity and infant care institutions offers an opportunity to the migrant mother to acculturate to the host society (Goguikian Ratcliff and Diaz-Marchand, 2019). Although childbirth does not give right to naturalization in Switzerland, a baby born in the host country may represent an anchor and a link between the migrant mother and the new society.

### Limitations of the study

Several limitations of the study need to be considered. First, documented migrant women in our sample were mostly highly educated women with a high socioeconomic status, which limits the generalizability of the results to all documented migrant women in Switzerland. The recruitment of migrant participants in the English-speaking antenatal classes lead to the overrepresentation of women working in international organizations. Furthermore, the choice of language groups (French, Spanish, Portuguese, or English) limited the inclusion of migrant women speaking other languages, such as documented women coming from non-Western societies. However, the languages chosen for this study are those spoken by most undocumented migrants in Switzerland, as the majority of them come from Latin American countries including Brazil. Furthermore, the relatively small sample size was due to the difficulty to recruit and keep migrant women who had no legal status. Factors hindering their participation included the fear of being denounced as undocumented, missing time due to high workload, or language problems.

Second, we used self-report questionnaires (EPDS, STAI) to measure depressive and anxiety symptoms. These are convenient and easy-to-use methods, but they are not appropriate for establishing a diagnosis of depression and anxiety. A psychiatric classification based, for example, on DSM-5 criteria would give a more accurate estimate of the rates of anxiety and depression disorders, but the establishment of diagnoses was beyond the aims of the present study. It is also worth noting that the antecedents of anxiety or depression disorders for the present sample were unknown.

It may be also argued that the self-report questionnaires used in the present study have been conceived in the Western scientific world and may not capture the non-Western patterns of expression of postpartum anxiety and depression symptoms. To reduce measurement errors due to cultural variables, validated versions of the questionnaires were used. In addition, all questionnaires were completed during oral interviews, therefore, in case of misunderstanding or ambiguity, the researcher was able to reformulate problematic items. The researcher’s physical presence could have affected the results by creating response bias, but the preceding clinical interview helped to establish a climate of trust and address anxiety and depressive symptoms before completing the questionnaires.

## Conclusion and implications

The present study showed that migration *per se* does not represent a risk factor for postpartum depression and anxiety. In fact, the novel feature of this study was to include advantaged migrant women. The results revealed that the latter were not at higher risk for postpartum anxiety and depression symptoms than Swiss women, whereas low-income undocumented migrant women showed high levels of such symptoms. Therefore, the results stress the negative impact of cumulated psychosocial difficulties on postpartum emotional distress. The study highlights the importance of considering migrant women’s legal status and living conditions when screening for postpartum anxiety and depression. Further, psychosocial follow-up programs should be put in place at the Maternity units for the most vulnerable women.

The results of this study should be replicated in different migrant groups living in other European or high-income countries, as the multicultural environment of Geneva is very specific and makes it difficult to generalize the findings to other cultural contexts. Furthermore, a group of low-income native women may be included in future studies to further inform about the particular role of environmental and migration-related factors influencing postpartum depression. Finally, it would be of great interest to study the mother–child relationship and bonding in the three groups, and its effects on child development.

## Data Availability

The raw data supporting the conclusions of this article will be made available by the authors, without undue reservation.
